# Phytoextract-mediated Cupper nanoparticles *via Acacia saligna*: synthesis, characterization and in vitro anticancer and apoptosis inducing effects

**DOI:** 10.1186/s40643-025-00918-0

**Published:** 2025-08-06

**Authors:** Fuad O. Abdullah

**Affiliations:** 1 Department of Chemistry, College of Science, Salahaddin University-Erbil, Erbil, Kurdistan Region Iraq; 2 Department of Pharmaceutical Chemistry, Faculty of Pharmacy, Tishk International University, Erbil, Kurdistan Region Iraq

**Keywords:** Phytoextract, CuO NPs, Anticancer, Apoptosis

## Abstract

Copper oxide nanoparticles (CuO NPs) have been utilized in various applications over the past few decades. This investigates the anti-cancer and apoptosis-inducing effects of green synthesis of copper oxide nanoparticles mediated by using *Acacia saligna* flower extract. The flower extract was mixed with a solution of copper sulphate pentahydrate (CuSO_4_·5H_2_O) and sodium hydroxide (NaOH) as a catalyst. UV-Vis spectroscopy, FTIR (Fourier Transform Infrared Spectroscopy), XRD (X-ray Diffraction), and SEM (Scanning electron microscope), EDS (Energy Dispersive Spectroscopy) and TEM (Transmission Electron Microscopy) analysis were conducted to characterize the synthesized CuO NPs. The average particle size of the NPs was found to be 8.488 nm. Additionally, the CuO NPs exhibited noteworthy anticancer activity against the MCF-7, PC3, HT-29, and U-87MG cell lines (18.11 ± 25, 8.92 ± 0.73, 19.48 ± 0.24, and 26.08 ± 0.57, respectively). In particular, the CuO NPs demonstrated a significant effect on the human colorectal adenocarcinoma cell line (HT-29) with an IC_50_ value of 19.48 ± 0.24 µg/mL, surpassing that of the standard (Cis-platin, 22.20 ± 0.72). The CuO NPs also induced apoptotic-mediated programmed cell death in cancer cell lines. In conclude, this investigation suggests that green-synthesized CuO NPs with *Acacia saligna* flower extract could serve as a viable alternative for anticancer applications in biomedicine. However, further research is needed to understand the detailed mechanisms of action and evaluate their in vivo efficacy to better establish their potential for clinical applications.

## Graphical Abstract

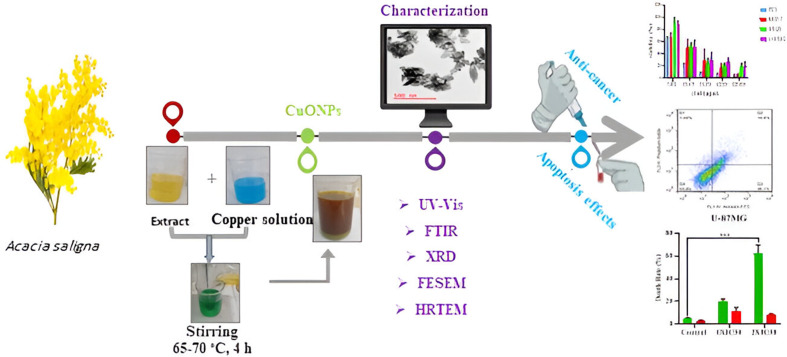

## Introduction

The green synthesis of nanoparticles has emerged as a promising alternative to conventional chemical and physical methods, offering a more sustainable, cost-effective, and environmentally friendly approach (Fierascu et al. 2019; Kharissova et al. 2013; Manzoor et al. 2018; Reddy et al. 2015). In recent years, plant-mediated synthesis has gained significant attention due to its scalability and ability to produce nanoparticles with enhanced biological compatibility (Ahmed et al. 2016b; Iravani 2011; Haleemkhan et al. 2015). Aqueous plant extracts are rich in diverse phytochemicals such as, phenolics, flavonoids, alkaloids, terpenoids, saponins, and steroids, that serve as both reducing and stabilizing agents during nanoparticle formation (Abdullah et al. 2016, 2018; Fuad et al. 2017; Hussain et al.). These biomolecules, particularly those with hydroxyl and carbonyl functional groups, play a vital role in metal ion reduction and surface stabilization, preventing aggregation and facilitating the formation of phyto-metal nanoparticles (PhM-NPs) with controlled size and morphology (Sajadi et al. 2018; Yugandhar et al. 2015; Yousaf and Saleh 2018; Nasrollahzadeh et al. 2015; Azizi et al. 2017). Compared to chemical synthesis, green methods offer superior biocompatibility and reduced toxicity (Ismail et al. 2016). Among various metal oxide nanoparticles, copper oxide nanoparticles (CuO NPs) have gained substantial interest due to their unique physicochemical properties and significant biological activities, including antimicrobial, antioxidant, and anticancer effects (Cookson 2012; Veisi et al. 2015; Duan et al. 2015; Sharmila et al. 2017; Shaik et al. 2017; Majumdar et al. 2017). The synthesis of CuO NPs using plant extracts has demonstrated considerable potential in pharmacological applications. Furthermore, employing non-toxic, renewable phytosources for CuO NP synthesis enhances surface stability and biocompatibility, making them suitable for biomedical applications, particularly in cancer therapy (Ngnie et al. 2016; Hussain et al. 2015; Majumdar et al. 2016).

In this study, we report the green synthesis of CuO nanoparticles using the flower extract of Acacia saligna, a plant known for its rich content of phenolic and flavonoid compounds (Kuo et al. 2012), These phytochemicals are anticipated to act as natural reducing and stabilizing agents during nanoparticle formation. The synthesized nanoparticles were characterized using various analytical techniques, including SEM, XRD, FT-IR, TEM, and UV-Vis (Assefa et al. 2024).

To the best of our knowledge, this is the first report utilizing *A. saligna* flower extract for the biosynthesis of CuO nanoparticles and evaluating their anticancer potential. The aim of this research is to develop an eco-friendly synthesis route for CuO NPs and to investigate their cytotoxic and apoptosis-inducing effects on human cancer cell lines.

## Experimental sections

### Material

Copper sulfate pentahydrate (CuSO_4_.5H_2_O) was purchased from Sigma-Aldrich (98%), sodium hydroxide (NaOH) was used to monitoring the pH, were received from Merck. Deionized water. The flowers of *Acacia saligna* have been collected in Erbil-Iraq. The dissolution of 10 g of CuSO_4_.5H_2_O in 400 mL deionized water yielded a 0.1 mM stock solution of CuSO_4_.5H_2_O. All of the chemical and solvents utilized were of analytical grade.

### Collection of plant material

The plant components were gathered in April of Erbil-Kurdistan region/Iraq. The plants were taking permission to collect and identified by the botanist and put on display in the Salahaddin University-Erbil/Iraq. The accession number for the voucher specimens (7821, Herbarium). *Acacia saligna* flowers were dried by air in a dark, comfortable environment without any light. The dried plants were then finely pulverized in a lab grinding-mill and sieved to produce a homogenous powder for examination. Then, in order to prevent contamination, it is kept in glass bottles in a dark environment.

### Preparation of the phytoextract

The dried plant materials (10 g) of the flowers of *Acacia saligna* mixed with deionized water (250 mL) were subjected to Microwave-assisted extraction (Panasonic P90N28AP-S3) at 800 W: 5 min were spent utilizing a 20-second interval irradiation cycle (Mohammed and Abdullah 2022). The deionized water was evaporated from the extracts using a rotary evaporator at 35–40 °C under vacuum. Then, to avoid contamination, it is stored in glass bottles in a dark area (Yilmaz 2020).

### Synthesis of CuO NPs

The synthesis of CuO NPs from *Acacia saligna* flowers extract solution. 10 g of CuSO_4_·5H_2_O was dissolved in 400 mL of Deionized water (DI) to initiate the green synthesis process for CuO NPs. After, 100 mg of *Acacia saligna extract* dissolved in 100 mL of deionized water then mixing with 100 mL of 0.1 mM CuSO_4_·5H_2_O solution, the pH was kept at 7.0 with NaOH. The solution then underwent to a reflux at a magnetic stirrer. The colour of the solution changed as it was stirring with a from green to a brown while maintaining for 4 h at 65–70 °C. After centrifuging the solution was filtered. The solid precipitate was washed three times with deionized water, followed by an 100% ethanol wash for CuO NPs separation, dried at 60 °C for 4 h, and kept at 4 °C for further application.

### Characterization of synthesized CuO NPs

An Oceanian Optics JAZ spectrophotometer (USA) was used to gather the UV-visible spectra of CuO nanoparticles that were successfully obtained. The formation of copper oxide nanoparticles in colloidal solution was seen at wavelengths between 200 and 800 nm in the UV spectrum. Utilizing KBr pellet, the FTIR spectrometer (Perkin Elmer, Spectrum-2, USA) was employed to gather functional group data within the 4000–400 cm − 1 range. The resulting CuO NPs’ FTIR spectra was studied. CuO NPs have been shown to exhibit distinct modes of vibration, which are utilized to assess the existence of several functional groups that support the extract of Acacia saligna flowers. Using Cu-Kα radiation (λ = 1.54 Å) and a Shimadzu XRD-6000 diffractometer running at 40 kV and 20 mA current, an XRD measurement of the CuO NPs was performed with just 5.0 ml of the extract added. 10° and 75° scanning range values were used to investigate and acquire the XRD spectra for CuO (Copper (II) oxide), the standard JCPDS card is typically: JCPDS No: 45–0937. The surface morphology of CuO NPs was investigated using a SEM (CARL ZEISS, Jena, Germany). Using an Acacia saligna extract, the interior morphology of the CuO nanoparticles was examined, and TEM (JEOL, JEM-2100, Japan) pictures were taken. To provide context, 5.0 ml of the materials were sonicated in ethanol, and a drop of the mixture was placed layer by layer on a copper grid containing 300-mesh carbon for magnetic measurements.

### MTT assay for anticancer activity

CuO NPs sample of cell viability were run against HT-29, PC3, U-87MG and MCF-7, cell lines. The cells were cultivated in a humidified atmosphere with 95% medium and 5% CO2 in RPMI 1640 (PC3), DMEM low glucose (U-87MG), and DMEM high glucose (MCF-7 and HT-29) media supplemented with 10% fetal bovine serum (FBS) and 1% (V/V) penicillin/streptomycin. The MTT method was used to assess cell survival. Lastly, each well of a 96-well plate containing 1.0 × 10^4^ cells was pre-cultured for 16 h in an incubator. Following this, the cells were exposed to various complex concentrations: 7.812, 15.675, 31.250, 62.500, and 125.000 µg/mL and Cis-platin as a standard drug concentration: 1.890, 3.790, 7.570, 15.150, and 30.300 µg/mL. in fresh media for a duration of 72 h. When the proper amount of time had elapsed, a new medium containing MTT solution with a final concentration of 0.50 mg/ml was added to each well. After that, it was incubated for a further four hours in the same conditions. Subsequently, the growth medium-containing solvent buffer was eliminated, and 100 µL of 100% DMSO was utilized to dissolve the crystal formazan. The formazan and background absorbance of the sample were then determined using the BMG Spectro Nano Elizabeth Reader at two wavelengths, 570 nm and 630 nm, respectively. The proportion of live cells was calculated using the formula shown below: Cell-viability (%) = [A_T (sample)_ / A_T (control)_] × 100, where the AT is defined as, A_570_ - A_630_. Using the GraphPad Prism 8 program, the IC_50_ concentration was calculated as the Mean Standard Deviation (STDEV) from three different tests (Jabbar et al. 2022).

### Apoptosis/Necrosis assay

A total of 2 × 10^5^ different cancer cell lines, such as PC3, MCF-7, HT-29, and U-87MG, were pre-cultured for 16 h before exposure to CuO NPs for 24 h. The test was performed according to the Bioscience TM Annexin V apoptosis detection kit (Invitrogen) staining protocols. After suspending the cells with trypsin 0.25%, cells were washed once with PBS to remove extra nanoparticles, followed by washing once with 1000 µL of 1X binding buffer. After that, the cells were left to float for a further fifteen minutes in a 1X binding solution that contained five microliters of Annexin V-fluorescein isothiocyanate (Annexin-FITC) in total volume of 100 µL of binding buffer. In the next step, cells were precipitated at 1200 RPM for 3 min and resuspended in 200 µL of the same buffer with 5 µL of a propidium iodide (PI) solution. Finally, using BD FACS Calibur TM flow cytometry (BD Biosciences, San Jose, CA, USA), the rates of apoptosis were determined by adding early and late apoptosis (Abdullah 2024).

## Results and discussions

### Characterization of synthesized nanoparticle (CuO NPs)

The change in the colour of the reaction solution suggests the synthesis of CuO NPs by the reduction of CuSO_4_·5H_2_O during treatment with extracts of *Acacia saligna* flowers. The change in color of the reaction solution after 2 h reveals the synthesis of CuO NPs. The result indicates that the Cu-Extract^2+^ ions in the reaction mixture have changed to copper oxide with nanometric size. In the synthesis of CuO NPs, different types of plant extracts are used as reducing and stabilizing agents. The results of nanoparticles have no surface instead of encased in a medium or gel, and their catalytic and other characteristics can be restricted, while particle stabilized and microgel stabilized nanoparticles characteristics may be altered by modifying the temperature and pH.

#### UV-vis spectroscopy

**Fig. 1:**
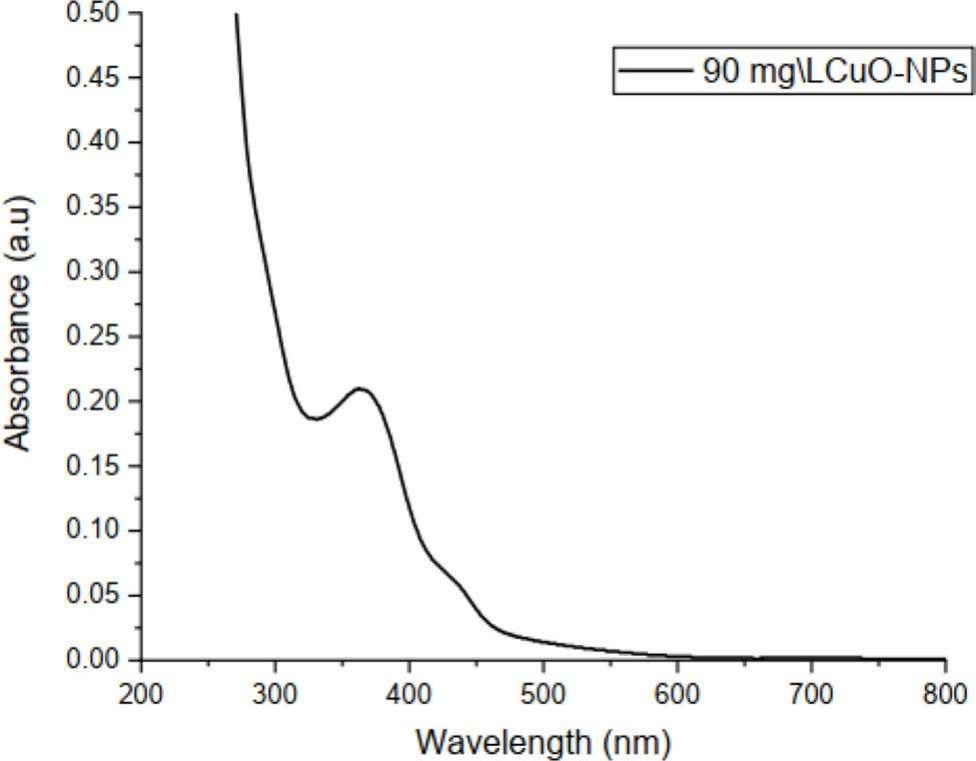
The UV visible spectra of a CuO NPs prepared by *A. saligna* flower extract

UV–visible spectroscopy was used to examine the CuO NPs in order to determine the optical band gap. It was discovered that there was a noticeable peak at 363 nm that might be related to surface plasmon resonance (SPR). The production of CuO NPs is shown by the SPR at 363 nm. The oscillation of the nanoparticles’ surface electron led to SPR. Mie’s hypothesis states that the form of the generated nanoparticles mostly determines the number of SPR bands. One SPR band is largely responsible for the nanoparticle’s spherical shape. The band gap energy comparable to the peak absorption wavelength was computed using the equation. The greatest absorption peak of the produced CuO nanoparticles, which reflects the blue shift absorption seen in Fig. 1, is seen at 363 nm. Thus, this finding supported previous study (Shashanka 2021).

#### FT-ATR spectral analysis

**Fig. 2:**
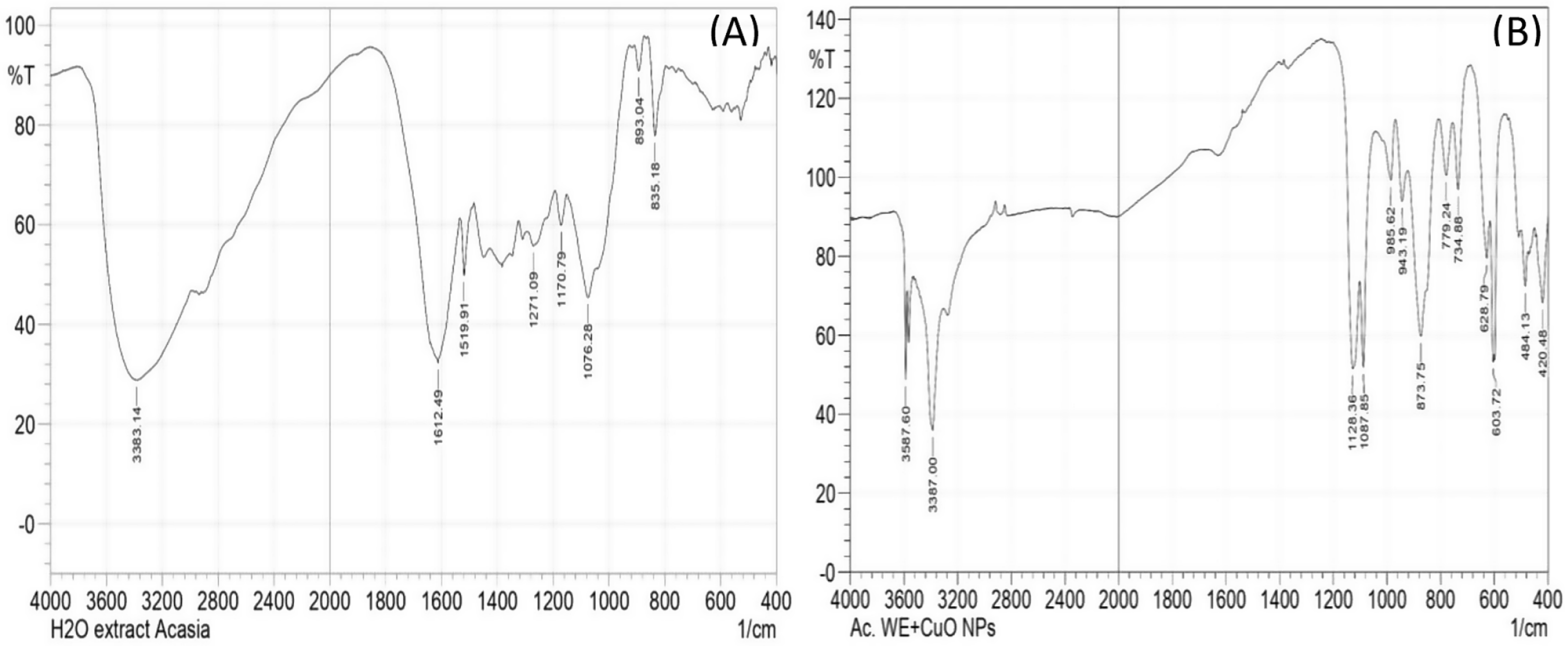
FTIR spectra of *A. saligna* H_2_O extract (**A**), and synthesized CuONPs by using *A. saligna* water extract (**B**)

The FT-ATR spectra of the aqueous *Acacia saligna* extract (Fig. 2A) and the synthesized copper oxide nanoparticles (CuO NPs) using the same extract (Fig. 2B) were analyzed in the range of 4000–400 cm⁻¹ to determine the functional groups involved in the bioreduction and stabilization processes (Dutta et al. 2019). In the spectrum of *A. saligna* extract (Fig. 2A), a broad absorption band at 3383 cm⁻¹ is attributed to O–H stretching vibrations, which are commonly associated with hydroxyl groups in phenols, flavonoids, and polyphenolic compounds (Mittal et al. 2013). These compounds are known to act as reducing and capping agents in the green synthesis of nanoparticles. A distinct band observed around 1641 cm⁻¹ corresponds to C = O stretching vibrations from carbonyl groups such as those in ketones, aldehydes, or carboxylic acids. These groups may originate from secondary plant metabolites and contribute to metal ion reduction by facilitating electron transfer (Al-Gburi 2018). Additional peaks at 1046 cm⁻¹ indicate C–O stretching of alcohols or ethers, which may also participate in nanoparticle stabilization through hydrogen bonding or coordination with copper ions (Ahmed et al. 2016a). After the synthesis of CuO NPs (Fig. 2B), noticeable changes occur in the FTIR spectrum, indicating interaction between the plant extract components and the nanoparticles. The broad peak at 3587 cm⁻¹ and 3387 cm⁻¹ signifies the continued presence of hydroxyl groups, suggesting their involvement in both reduction of Cu²⁺ to CuO and surface functionalization of the resulting nanoparticles (Nasrollahzadeh et al. 2021). The shift and broadening of the hydroxyl peak reflect hydrogen bonding between the plant-derived compounds and the nanoparticle surface. Importantly, new peaks appear at 484 cm⁻¹ and 420 cm⁻¹, which are assigned to Cu–O stretching vibrations, confirming the successful formation of copper oxide nanoparticles (Lateef et al. 2019; Assefa et al. 2024). The appearance of these metal-oxygen bands validates the transformation of copper ions into CuO in the presence of the A. saligna extract. The shifts in peak positions (e.g., from 1046 to 1025 cm⁻¹ and from 1641 to ~ 1630 cm⁻¹) between the extract and CuO NP spectra further indicate the chemical interaction and complexation of plant phytochemicals with copper ions during synthesis, which plays a crucial role in both reduction and nanoparticle stabilization.

#### The scanning electron microscope (FESEM)

**Fig. 3:**
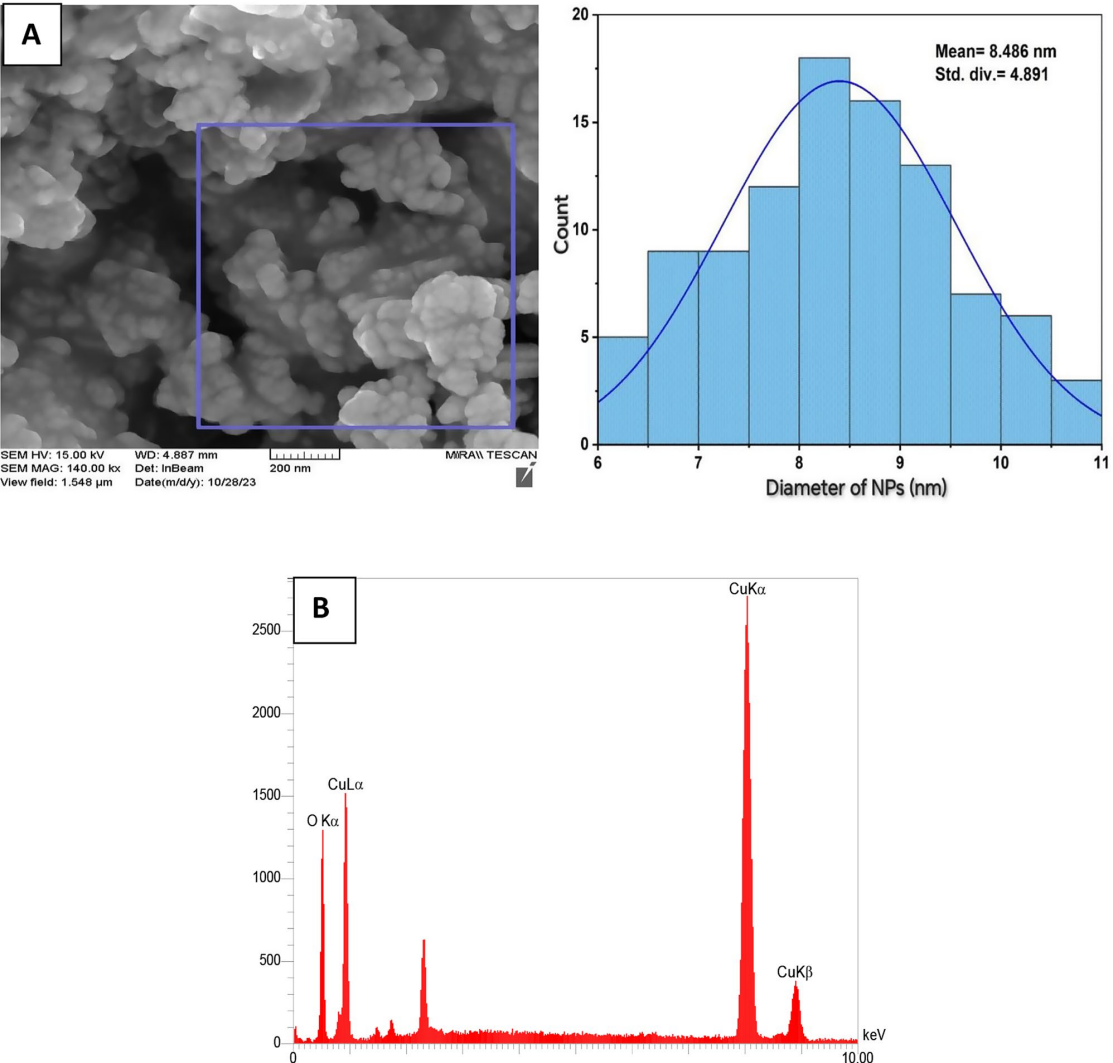
Electron micrographs (FE-SEM) with particle size distribution (**A**) and EDS image (**B**) of CuONPs

The morphology and particle size of the synthesized CuO nanoparticles (NPs) were characterized using Field Emission Scanning Electron Microscopy (FESEM), as shown in Fig. 3A. The FESEM images confirm that the green-synthesized CuO NPs possess a predominantly spherical morphology with slight surface roughness and a tendency to form clusters. The particles appear well-distributed, though some degree of agglomeration is observed, which is common in nanoscale materials due to high surface energy and van der Waals interactions. The average particle size was estimated to be approximately 8.488 nm based on the particle size distribution curve, indicating successful synthesis at the nanoscale, consistent with previous reports (Kannan et al. 2022). The observed agglomeration is likely driven by the particles’ efforts to minimize surface free energy, a phenomenon frequently reported in nanoparticle systems. As particle size increases, the specific surface area decreases, which can lead to reduced agglomeration, as also noted in this study. Additionally, the relatively uniform particle size and shape support the effectiveness of the green synthesis method in controlling nanoparticle morphology (Priya et al. 2023). The elemental composition of the CuO NPs was determined using EDS, as depicted in Fig. 3B. The spectrum reveals prominent peaks corresponding to copper (Cu) and oxygen (O), confirming the presence of CuO. Quantitative analysis indicates that the nanoparticles contain approximately 62.21% copper and 37.79% oxygen by weight, which is consistent with the stoichiometry of CuO. The absence of additional elemental peaks suggests high purity of the synthesized nanoparticles and supports the efficiency of the green synthesis approach.

The strong Cu and O signals further validate the formation of copper oxide nanoparticles, particularly in their oxidized state (Cu²⁺), which is crucial for various catalytic and antimicrobial applications. The green synthesis method, employing plant-based or eco-friendly agents, not only facilitates nanoparticle formation but also contributes to the environmental sustainability of the process.

#### X-ray diffraction analysis (XRD)

**Fig. 4:**
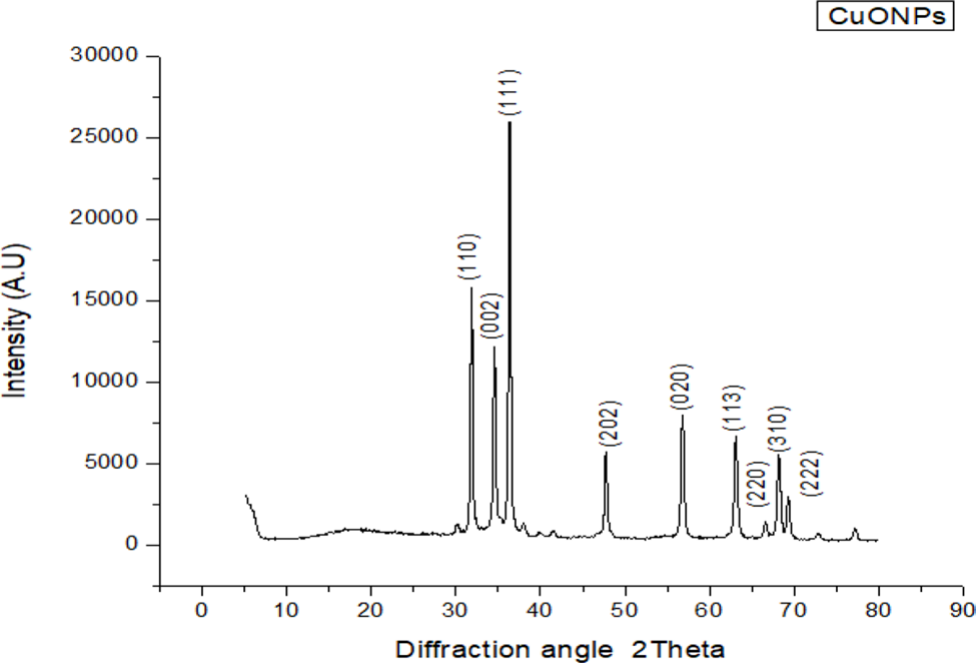
XRD diffraction pattern of a CuO nanoparticles synthesized from *A. saligna* flower extract

X-ray diffraction (XRD) analysis was performed to investigate the crystalline nature and phase purity of the biosynthesized copper oxide nanoparticles (CuO NPs). The obtained diffraction pattern is presented in Fig. 4, covering a 2θ range from 20° to 80°. The sharp and well-defined diffraction peaks indicate the formation of crystalline CuO nanoparticles. The most intense diffraction peaks were observed at 2θ values of 31.9°, 34.6°, and 36.4°, which correspond to the (110), (002), and (111) crystal planes, respectively. Additional peaks appearing at 2θ = 46.1°, 49.7°, 58.3°, 61.5°, 66.2°, and 68.0° were indexed to the (202), (020), (113), (220), (310), and (222) planes. These diffraction planes are consistent with the monoclinic phase of CuO, and all peak positions match well with the standard data from JCPDS Card No. 01-089-5899. The presence of only monoclinic CuO reflections with no secondary peaks suggests that the synthesized nanoparticles are of high phase purity and free from other copper-based impurities such as Cu₂O or metallic Cu. This confirms the successful biosynthesis of phase-pure CuO nanoparticles using Acacia saligna extract. The peak broadening observed in the XRD spectrum, particularly around the dominant reflections, is characteristic of nanoscale crystallite sizes. The average crystallite size (D) was estimated using the Scherrer equation: (Shashanka 2021).

$$D = \frac{{K\lambda }}{{\beta \cos \theta }}$$

where:

- D is the crystallite size,
- K is the shape factor (typically 0.9),
- λ is the X-ray wavelength (1.5406 Å for Cu Kα),
- β is the full width at half maximum (FWHM) of the peak in radians,
- θ is the Bragg diffraction angle.

#### Transmission electron microscopy (TEM) analysis

**Fig. 5:**
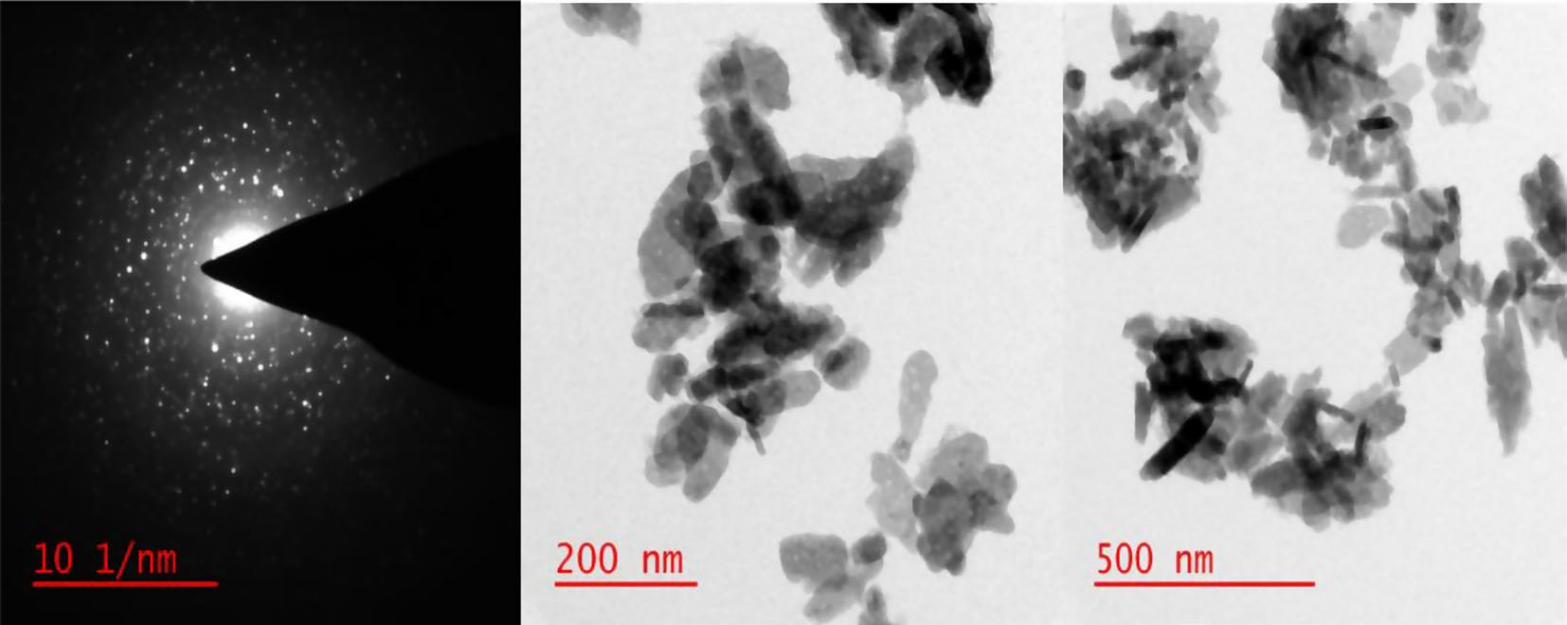
HRTEM images of copper oxide nanoparticles prepared by using *A. saligna* flowers extract

Figure 4 displays the HRTEM pictures of the produced CuO nanoparticles. *Acasia saligna* mediated CuO NPs were investigated for particle size and surface morphology using HRTEM (Dutta et al. 2022). The findings indicated that the CuO were cylindrical and polydisperse on the HRTEM images, as shown in Fig. 5, which may have been related to the presence of various bioactive phytochemical types. On the pictures, geometrical formations were discovered that indicated the successful interaction between copper ions and atoms and phytochemicals (Murthy et al. 2021).

### Anti-cancer activity

**Fig. 6:**
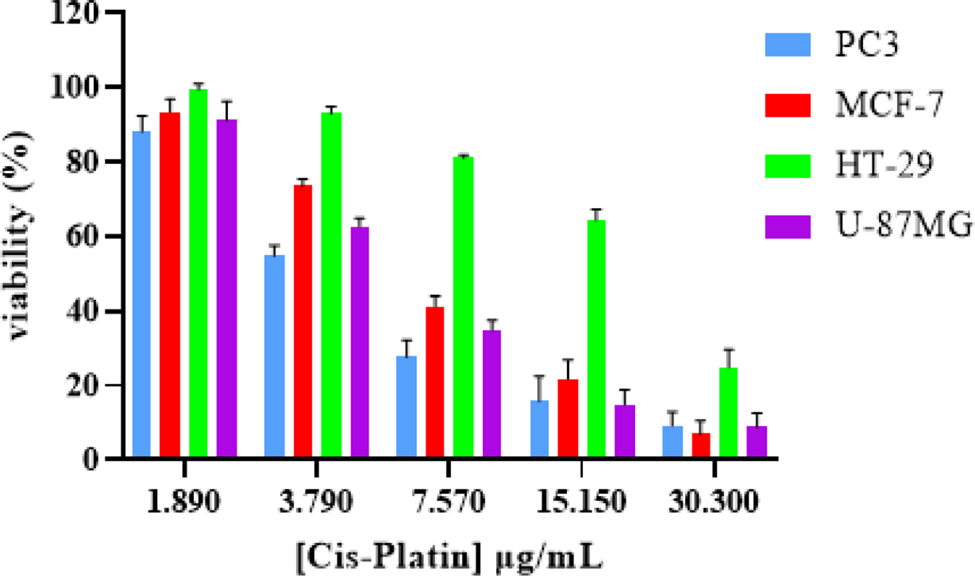
The percentage of viability of PC3, MCF-7, HT-29, and U87-MG cell lines after treatment with concentration cis-platen

**Fig. 7:**
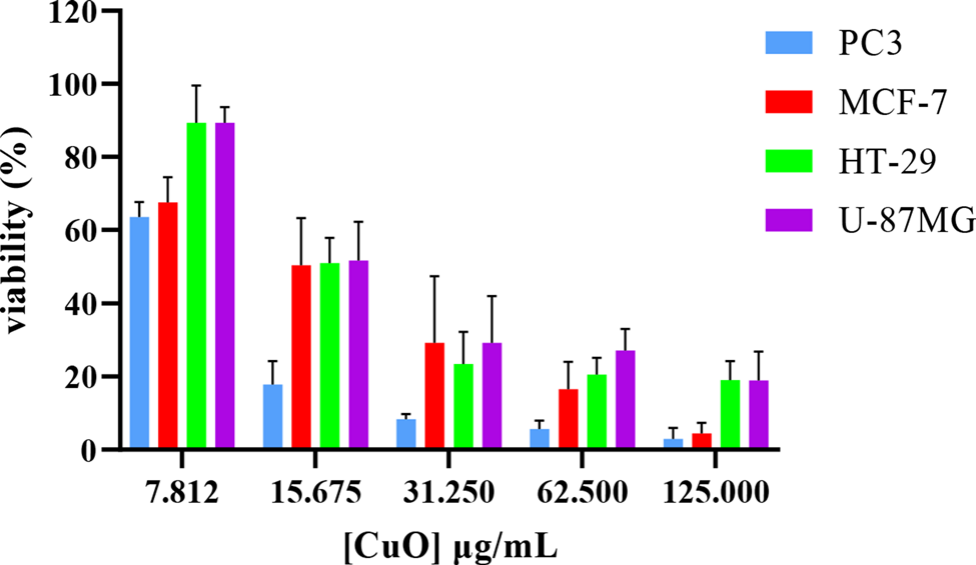
The percentage of viability of PC3, MCF-7, HT-29, and U87-MG cell lines after treatment with different concentrations of CuO NPs

The results are shown in (Table 1) of anticancer activity of the CuONPs synthesized by using *Acacia saligna* water extract against four different cell lines such as, PC3 (human prostate cancer), MCF-7 (human breast cancer), HT-29 human colon adenocarcinoma), and U87-MG (human glioblastoma) cell lines. In the present study, a significant and even more effect than Cis-platin (Fig. 6) was found in the human colon adenocarcinoma, HT-29 (*IC*_*50*_, 19.48 ± 0.24 (µg/ml), but for Cis-Platin (*IC*_*50*_, 22.20 ± 0.72 (µg/ml). The inhibitor’s efficacy is indicated by the *IC*_*50*_ value, where increased potency is indicated by lower *IC*_*50*_ values (Sebaugh 2011). According to the literature plant-based CuO NPs are advantageous due to the natural phytocompounds from the plant extract, which can enhance the therapeutic effects (Letchumanan et al. 2021; Aziz et al. 2023). A recent review study (Gebreslassie and Gebremeskel 2024) reported that CuO nanoparticles (CuONPs) were tested against various anticancer cell lines; however, only results for the MCF-7 cell line were provided, with IC₅₀ values ranging from 7.5 to over 100 µg/mL. Additionally, in this study, the CuO nanoparticles were tested on three new cell lines, which were evaluated for the first time. The anticancer activity of CuONPs on HT-29 was much more sensitive compared to other cells. The results also more effect to against other cancer cell lines, the order effects from higher to lower: PC3 (with *IC*_*50*_ value of 8.92 ± 0.73 µg/mL), was more effected compared to MCF-7 (with *IC*_*50*_ value of 18.11 ± 0.20 µg/mL), and moderate effect U87-MG (with *IC*_*50*_ value of 26.08 ± 0.57 µg/mL) cell lines as shown in Fig. 7.

**Table 1:** Anticancer activity of the CuO NPs against PC3, MCF-7, HT-29, and U87-MG cell lines

Sample	IC_50_ (µg/ml) ± SD			
	PC3	MCF-7	HT-29	U87-MG
CuO NPs	8.92 ± 0.73	18.11 ± 0.20	19.48 ± 0.24	26.08 ± 0.57
Cis Platin	4.85 ± 0.32	6.48 ± 0.26	22.20 ± 0.72	4.30 ± 0.233

### Apoptosis analysis

Apoptosis is a controlled cell death process that avoids triggering inflammatory responses, unlike necrosis (Aziz et al. 2025). To assess the apoptotic potential of the green-synthesized CuO nanoparticles (CuO NPs), Annexin V-FITC/Propidium Iodide (PI) double staining was performed using flow cytometry. This method differentiates early apoptotic (Annexin V⁺/PI⁻), late apoptotic (Annexin V⁺/PI⁺), and necrotic (Annexin V⁻/PI⁺) cell populations.

**Fig. 8:**
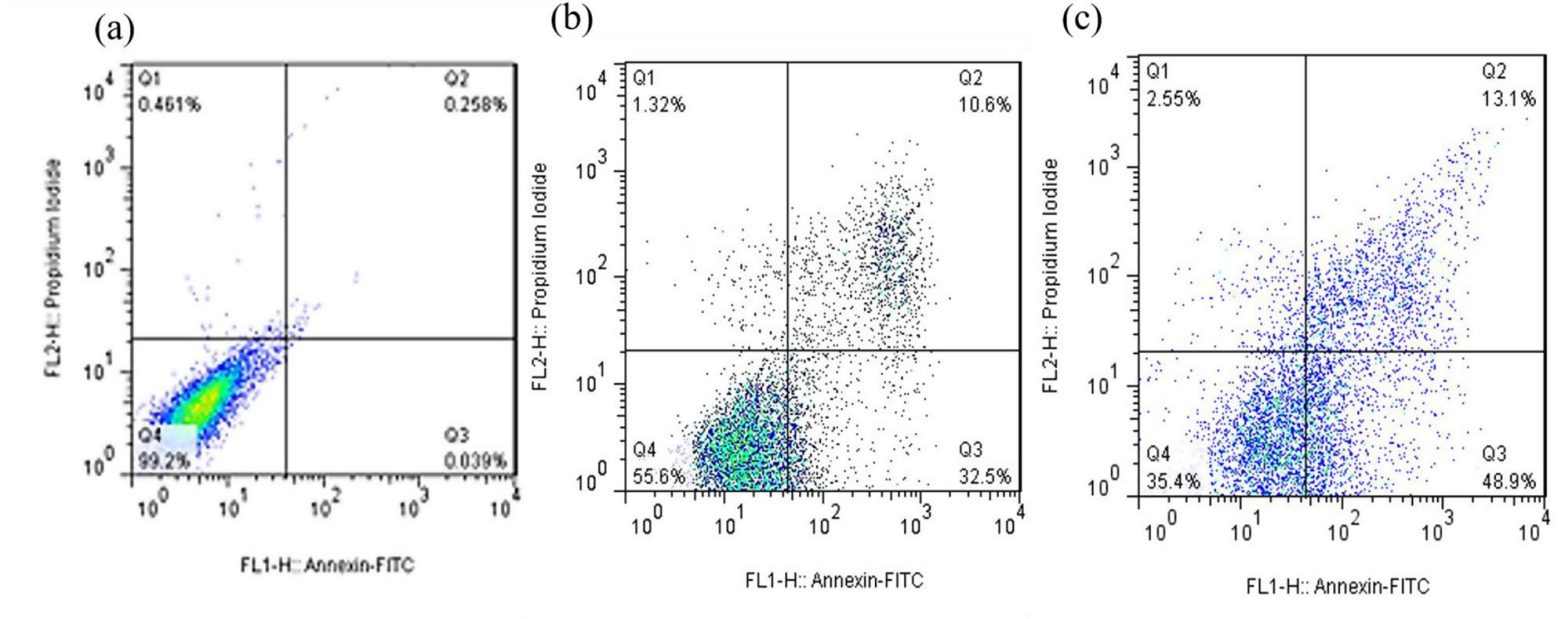
The demonstrative dot plots apoptosis/necrosis assay of PC3 cells after exposure to increasing concentrations of CuO NPs. **a**) Control cells, **b**) 8.9 µg/ml of CuONPs, and **c**) 17.8 µg/ml of CuO NPs

**Fig. 9:**
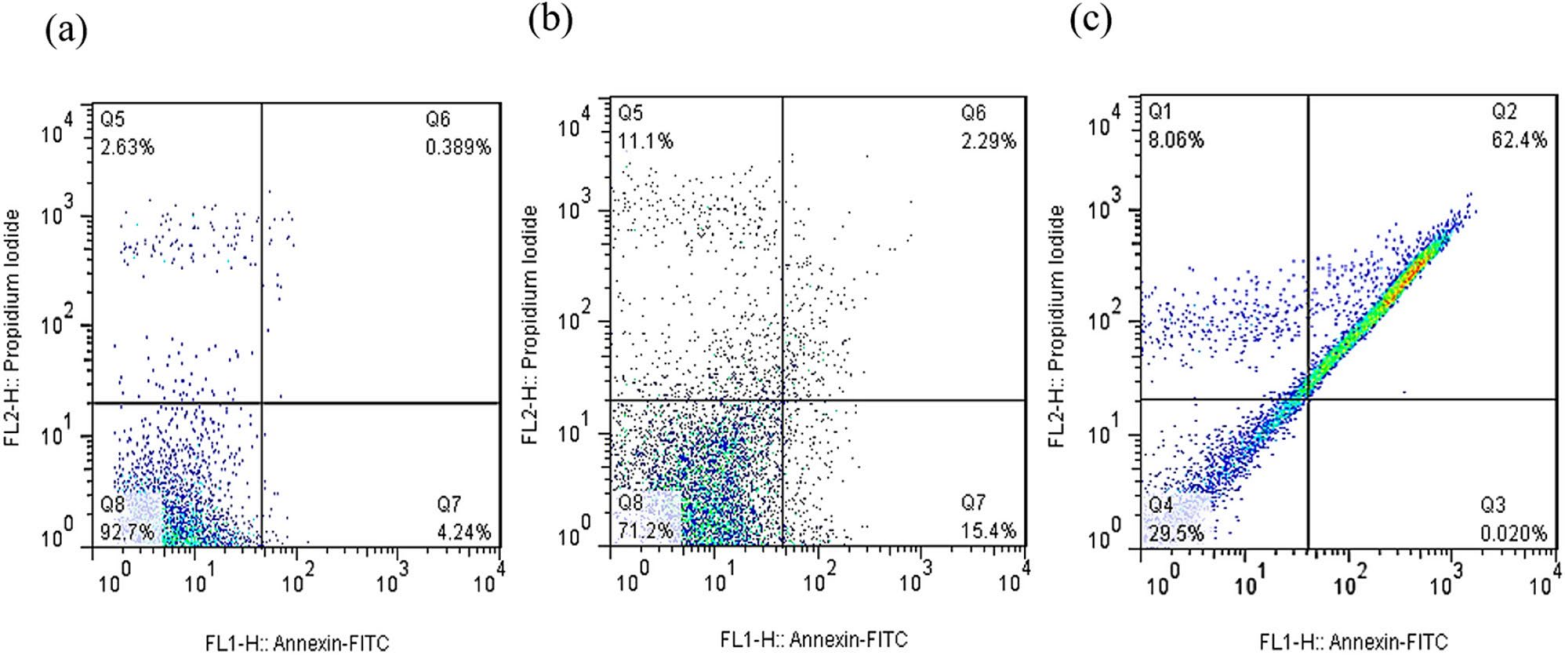
The demonstrative dot plots apoptosis/necrosis assay of U-87MG cells after exposure to increasing concentrations of CuO NPs. **a**) Control cells, **b**) 26.0 µg/ml of CuO NPs, and **c**) 52.0 µg/ml of CuO NPs

**Fig. 10:**
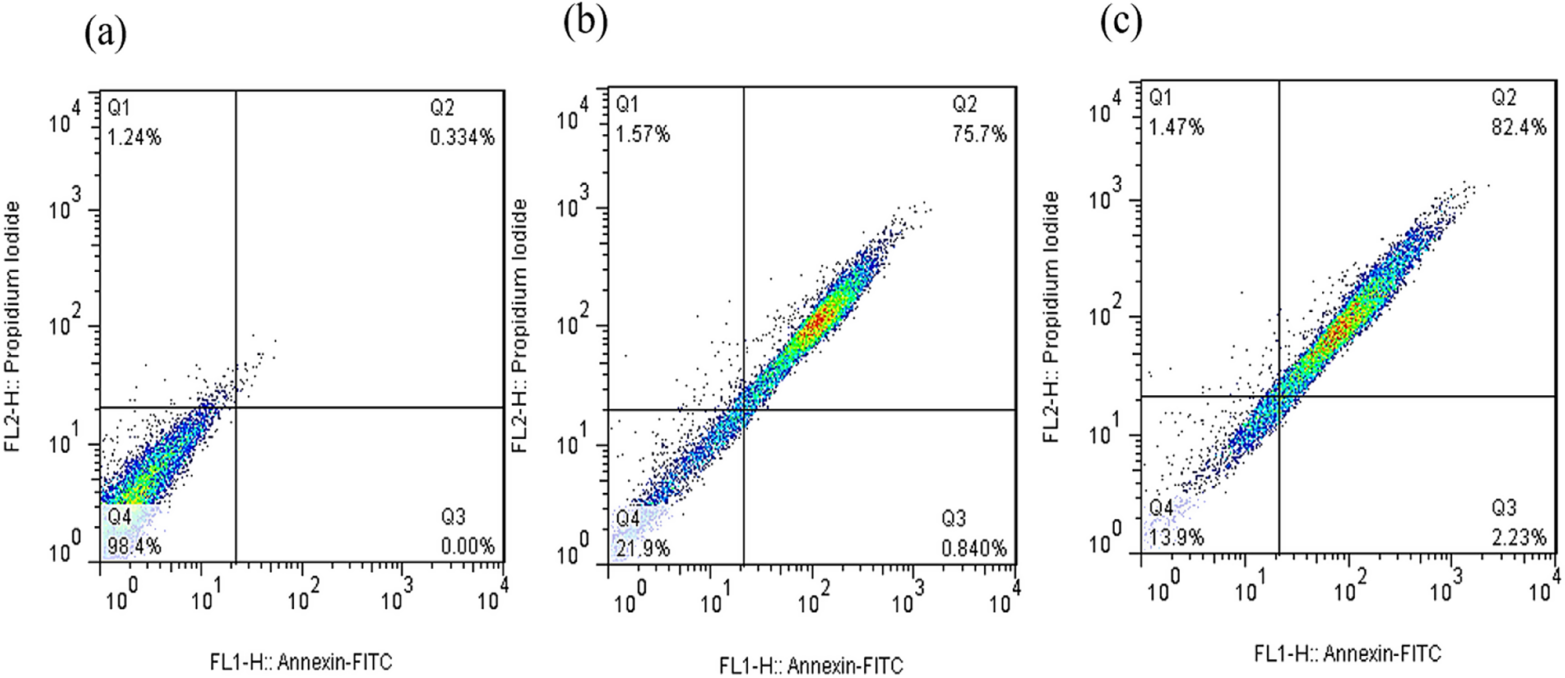
The demonstrative dot plots apoptosis/necrosis assay of MCF-7 cells after exposure to increasing concentrations of CuO NPs. **a**) Control cells, **b**) 18.1 µg/ml of CuO NPs, and **c**) 36.2 µg/ml of CuO NPs

**Fig. 11:**
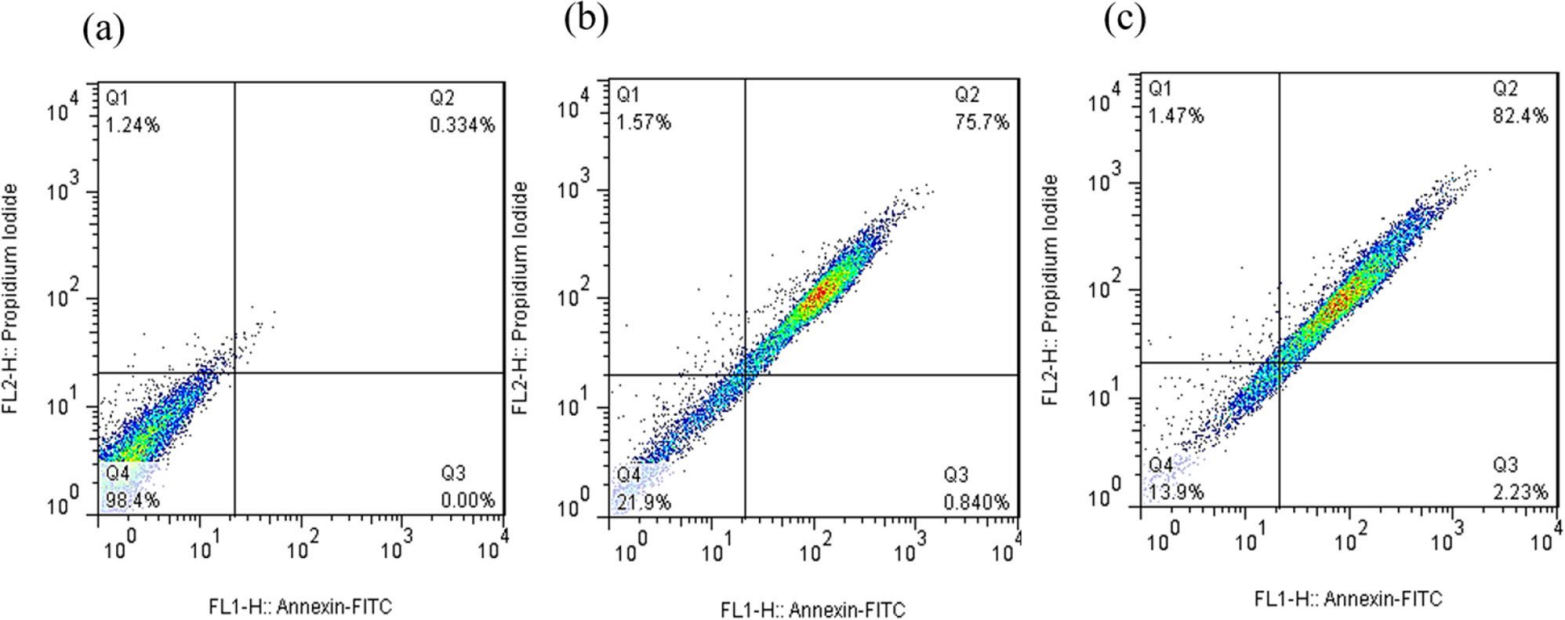
The demonstrative dot plots apoptosis/necrosis assay of HT-29 cells after exposure to increasing concentrations of CuO NPs. **a**) Control cells, **b**) 19.5 µg/ml of CuO NPs, and **c**) 39.0 µg/ml of CuO NPs

**Fig. 12:**
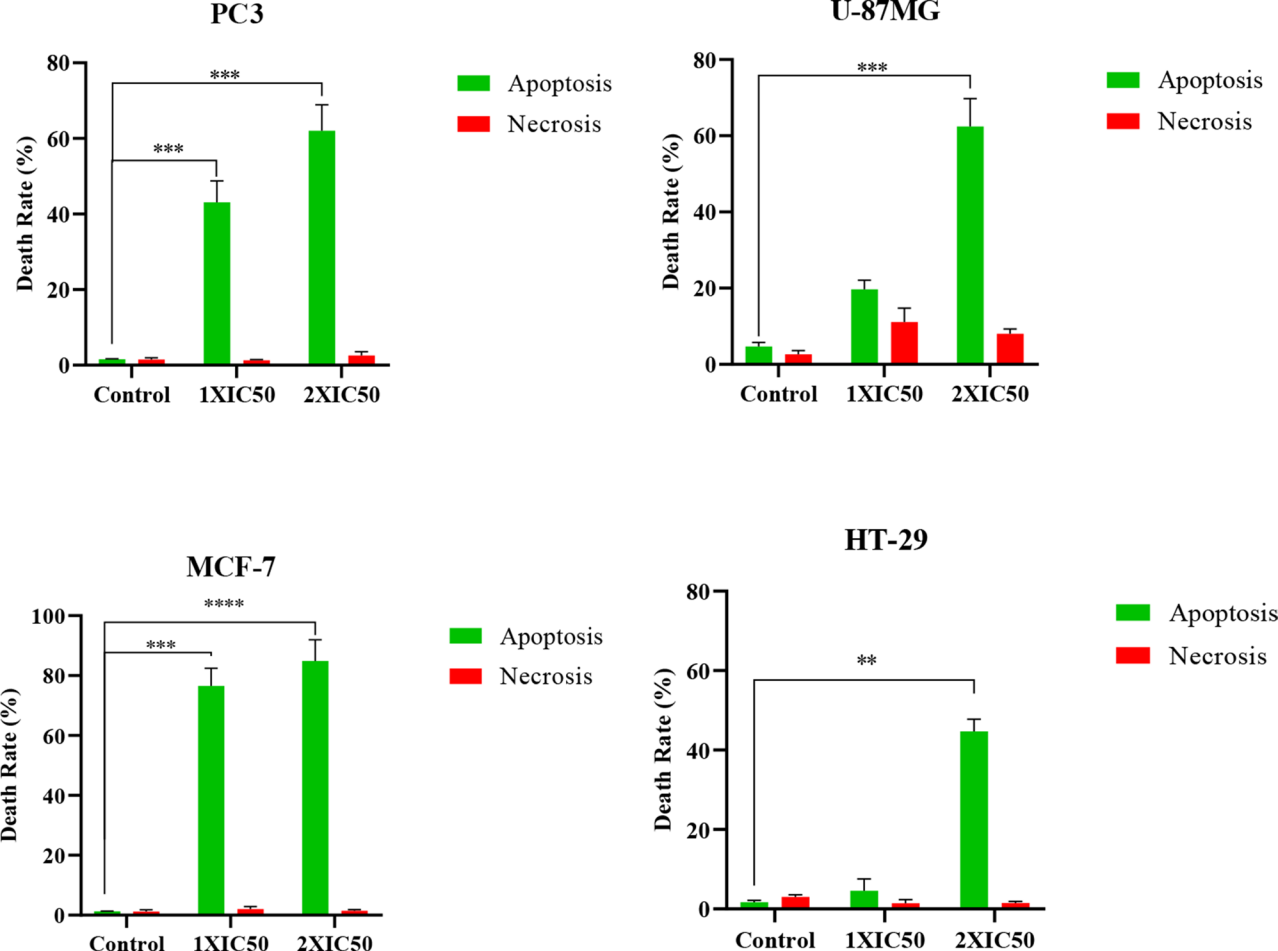
The apoptosis/necrosis rate of CuO NPs on PC3, U-87MG, MCF-7, and HT-29 cell lines. ​ The results obtained from three independent experiments were analyzed, and the statistical significance of the results was denoted by asterisks (∗∗ for *p*-value < 0.01; ∗∗∗ for *p*-value < 0.001 and ∗∗∗∗ for *p*-value < 0.0001) compared to the control group

The results showed (Figs. 8, 9, 10 and 11) that CuO NPs induced apoptosis in a concentration-dependent manner across all tested cancer cell lines (PC3, MCF-7, HT-29, and U-87MG). Notably, PC3 cells demonstrated the highest sensitivity to CuO NP treatment. At 2×IC₅₀ concentration, PC3 cells exhibited a significantly increased total apoptotic population (early + late) (*p* = 0.0006), with minimal necrosis observed. Similarly, significant apoptosis was observed in U-87MG (*p* = 0.0003), MCF-7 (*p* < 0.0001), and HT-29 (*p* = 0.006) cells at the same concentration (Fig. 12). According to Table 2, PC3 cells primarily underwent early apoptosis at higher CuO NP concentrations, whereas MCF-7, HT-29, and U-87MG cells were more inclined toward late-stage apoptosis. These findings suggest that while apoptosis is the predominant mechanism of cell death induced by CuO NPs in all cell lines, the timing and extent of apoptotic progression vary depending on the cell type.

Overall, the apoptosis assay corroborated the cytotoxicity results, indicating that the green-synthesized CuO NPs exert anticancer effects predominantly through the induction of apoptosis, with the response being both concentration- and cell-type-dependent.

**Table 2:** The percentage of cell populations in different stages (Early apoptosis, late apoptosis, and Necrotic)

Cell lines	*P*	Necrosis	Early Apoptosis	Late Apoptosis
**PC3**	CControl	0.461	0.039	0.258
	[CuO] = 8.9 µg/ml	1.32	32.5	10.6
	[CuO] = 17.8 µg/ml	2.55	48.90	13.1
**U-87MG**	CControl	2.63	4.24	0.389
	[CuO] = 26.0 µg/ml	11.10	15.40	2.29
	[CuO] = 52.0 µg/ml	8.06	0.02	62.40
**MCF-7**	CControl	1.24	0.00	0.334
	[CuO] = 18.1 µg/ml	1.57	0.84	75.70
	[CuO] = 36.2 µg/ml	1.47	2.23	82.40
**HT-29**	Control	3.00	0.39	0.21
	[CuO] = 19.5 µM	1.04	1.44	2.83
	[CuO] = 39 µM	1.48	16.6	26.1

## Conclusion

In conclude, validated for the study of green synthesized CuONPs from *A. saligna* flowers water extract. This plant (*A. saligna*) contains various bioactive phytochemicals such as, Phenolic and flavonoid compounds in the water extract were analyzed using HPLC: benzoic acid, caffeine, and *o*-coumaric acid were the most abundant phenolic compounds; while the flavonoid compounds naringenin, quercetin, and kaempferol were identified (Al-Huqail et al. 2019). These phytochemical have a vital role to reducing, stabilizing, and capping agents present in plant extracts and leading to the formation of biocompatible nanoparticles. The synthesized CuO nanoparticles exhibit desirable physicochemical properties, including controlled size, shape, and crystallinity, which are crucial for their biomedical applications. In this study CuONPs have significant effect for the human colon adenocarcinoma, HT-29 (*IC*_*50*_, 19.48 ± 0.24 (µg/ml) even better activity than cisplatin (standard). The total apoptosis rate early and late induced by CuO nanoparticles was significantly increased in PC3, U-87MG, and MCF-7 cells after treatment with a 2xIC50 concentration (*p* = 0.0006, *p* = 0.0003, and *p* < 0.0001, respectively), with no statistically significant necrosis observed. Similarly, HT-29 cells treated with the 2xIC50 concentration showed a significant increase in apoptosis (*p* = 0.006). Future work should focus on optimizing synthesis parameters, understanding the detailed mechanisms of action, and evaluating in vivo efficacy to further establish their potential for clinical applications.

## Data Availability

On demand, data will be provided.
